# Changing epidemiology of parvovirus B19 in the Netherlands since 1990, including its re-emergence after the COVID-19 pandemic

**DOI:** 10.1038/s41598-024-59582-7

**Published:** 2024-04-26

**Authors:** Anne Russcher, Michiel van Boven, Elisa Benincà, E. J. T. (Joanne) Verweij, Marijke W. A. Molenaar-de Backer, Hans L. Zaaijer, Ann C. T. M. Vossen, Aloys C. M. Kroes

**Affiliations:** 1 LUCID Medical Microbiology and Infection Control, Leiden University Medical Center, Leiden, The Netherlands; 2 Centre for Infectious Disease Control, National Institute for Public Health and the Environment (RIVM), Bilthoven, The Netherlands; 3 Julius Center for Health Sciences and Primary Care, Department of Epidemiology, University Medical Center Utrecht, Utrecht, The Netherlands; 4 Department of Obstetrics, Division of Fetal Therapy, Leiden University Medical Center, Leiden, The Netherlands; 5 Department of Blood-Borne Infections, Donor Medicine Research, Sanquin Blood Supply Foundation, Amsterdam, The Netherlands

**Keywords:** Epidemiology, Virology, Viral epidemiology, Clinical microbiology

## Abstract

Parvovirus B19V (B19V) infection during pregnancy can be complicated by potentially life-threatening fetal hydrops, which can be managed by intrauterine transfusion (IUT). This study investigates the long-term temporal patterns in the epidemiology of B19V and evaluates the impact on fetal hydrops, by combining data on B19V infections from the Dutch Sentinel Surveillance system in the period 1990 to 2023, Dutch blood banking data and hospital data on fetal hydrops. Using wavelet analysis, we identified annual epidemic cycles in the Netherlands in the period 1990–2019 and we identified superimposed multiannual cycles in the period 1990–2009. After 2009, no multiannual cycle could be identified, although the incidence fluctuated and correlates with number of IUT performed. As of 2020, weekly reports of B19V infection demonstrated a historically low incidence and B19V-DNA positive blood donors were nearly absent. From May 2020 to May 2023, no IUT for B19V-related hydrops was performed. In the spring of 2023, B19V infections re-emerged, reaching pre-pandemic epidemic levels. Due to the changes in B19V epidemiology over the last 30 years and the near-absence of B19V during the COVID-19 pandemic, the resulting low immunity levels may lead to rebound outbreaks. Alertness to severe complications such as fetal hydrops is warranted.

## Introduction

Parvovirus B19 (B19V) is best known as the pathogen involved in fifth disease, a self-limiting febrile erythematous illness in children. B19V infects erythrocyte progenitor cells, causing an arrest in erythropoiesis. In normal hosts, this arrest is temporary and does not lead to clinically significant anemia. However, the arrest of erythropoiesis may lead to severe anemia in susceptible persons such as immunocompromised hosts or individuals with a short half-life of red blood cells. When non-immune women acquire B19V infection during pregnancy, the virus can be transmitted to the fetus and fetal infection can lead to severe fetal anemia which in turn may lead to potentially fatal fetal hydrops. Fetal hydrops is managed by intrauterine erythrocyte transfusion (IUT), a highly specialized and costly procedure, which is only performed in specialized prenatal care hospitals. Insight in B19V epidemiology is relevant in view of the serious consequences of the infection in vulnerable hosts, in particular unborn children.

B19V infections occur according to a seasonal cycle with annual epidemics of varying size occurring in the spring^1,2^. Larger epidemics have been shown to occur once every four years, indicated as ‘oscillation’^1–4^. In the Netherlands, this pattern was last described in 2002, covering the period 1990–2002^1^. Data from other countries indicate that this pattern may vary; for example a six-year epidemic cycle is reported in Ireland^5^. Recently, it became clear that COVID-19 restrictions had a profound impact on the occurrence and seasonal variation of many viruses transmitted by the respiratory route, such as influenza virus and RSV^6^. The transmission dynamics of B19V also appeared to be affected by COVID-19 restrictions. B19V infections among blood donors fell sharply and became a rare finding in blood banks since the spring of 2020^7,8^.

The aim of this study was to describe the regular seasonal variation of B19V infections in the Netherlands over the past three decades, and to analyse the impact of the COVID-19 pandemic on B19V epidemiology, also considering possible clinical consequences such as fetal hydrops. To gain a comprehensive insight, we used data from the Dutch Sentinel Surveillance system, which receives data from laboratories on laboratory-confirmed B19V infection, combined with data from the Dutch national blood bank and hospital data on severe fetal B19V infection. Using wavelet analysis, an advanced technique to uncover periodicities in a time series, we show that the earlier described oscillation has become disturbed over the past decade, possibly due to a decreasing birth rate. After near-absence of B19V during the COVID-19 pandemic, B19V is now reemerging, which requires vigilance for severe complications in fetuses or other hosts susceptible to severe infection.

## Results

### Seasonal pattern and periodicity

**Figure 1:**
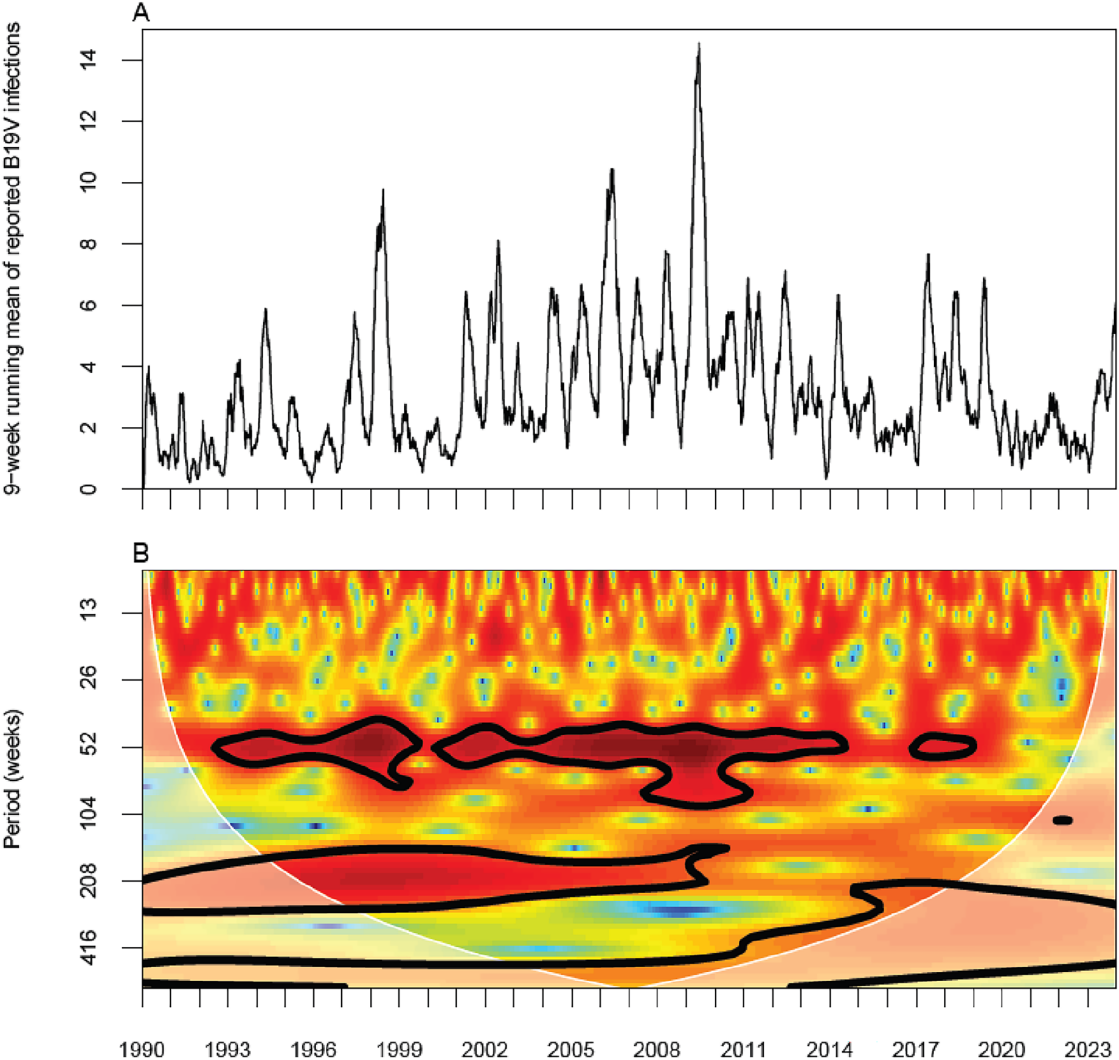
B19V epidemiology over a period of 33 years: (**A**) 9-week running mean of B19V-infections reported to the Dutch weekly Sentinel Surveillance system for the period Jan 1st, 1990–Dec 31st, 2023. (**B**) Wavelet power spectrum of the Sentinel Surveillance data of B19V infections for the period Jan 1st, 1990–Dec 31st, 2023. Colours indicate wavelet power (from low power in blue to high power in red). Black contour lines enclose significant areas (95% confidence) where the power is higher than the power of red noise with the same autocorrelation coefficient as the data. The shaded area represents the cone of influence, which is a region where edge effects become important. Significant periodicities are visible around 52 weeks (annual peaks) and around 208 weeks (four-year cycle) up to 2009.

**Figure 2:**
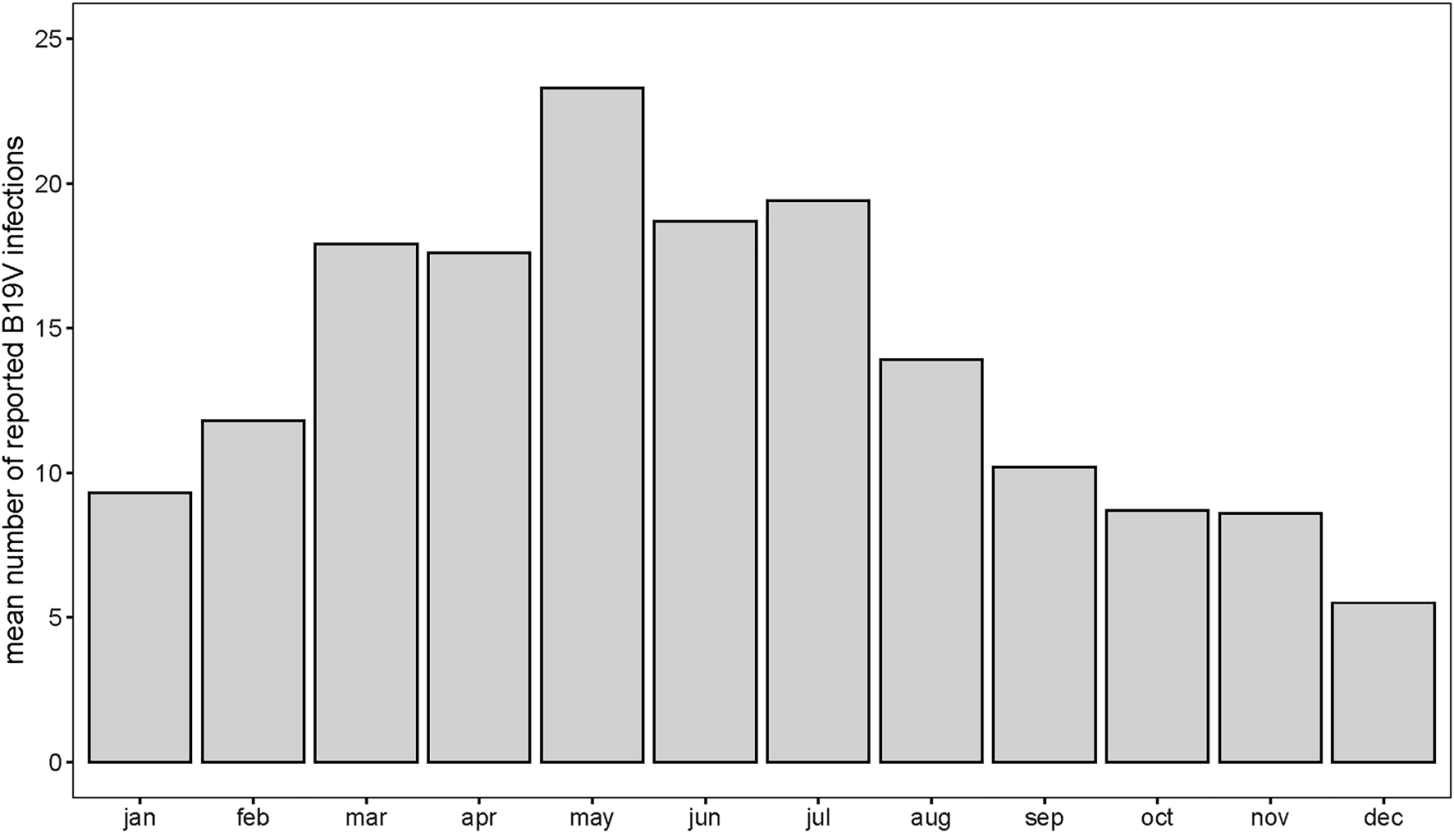
Seasonal variation in B19V incidence: Mean number of B19V infections per week reported to the national Sentinel Surveillance system for the period Jan 1st, 2002–Dec 31st, 2023.

Figure 1 (panel A) shows the 9-week running mean of the number of B19V infections based on national Sentinel Surveillance data for the period January 1st, 1990, to December 31st, 2023. For the period up to 2020, annual peaks are present, although the annual peaks are less pronounced in 2015 and 2016. Wavelet analysis confirmed this annual peak and showed that in the period 2020–2022, the annual peaks had disappeared (Fig. 1, panel B). For the period up to 2009, a 4-year periodic pattern is present in addition to the annual peaks. After 2009, a multiannual periodicity could not be identified by wavelet analysis. However, the incidence did fluctuate in the period 2010–2019, with the lowest number of infections reported in 2016 (n = 94) and the highest in 2010 (n = 221). Figure 2 shows that the annual peaks occur mostly in spring, with most infections being reported in May.

**Figure 3:**
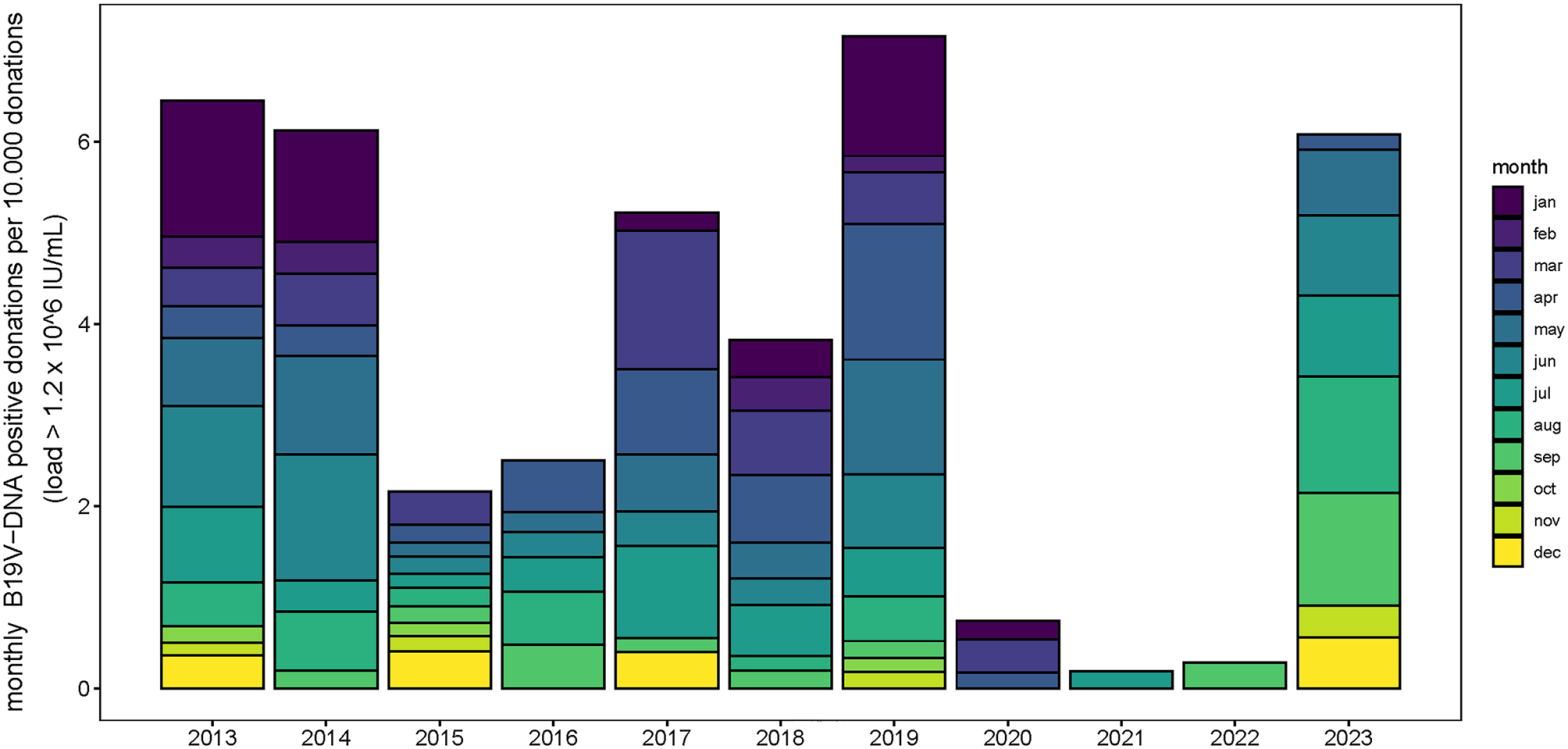
B19V positive blood donations: Number of monthly high-load B19V-DNA positive donations per 10.000 donations at the Dutch national blood bank for the period Jan 1st, 2013–Dec 31st, 2023.

**Figure 4:**
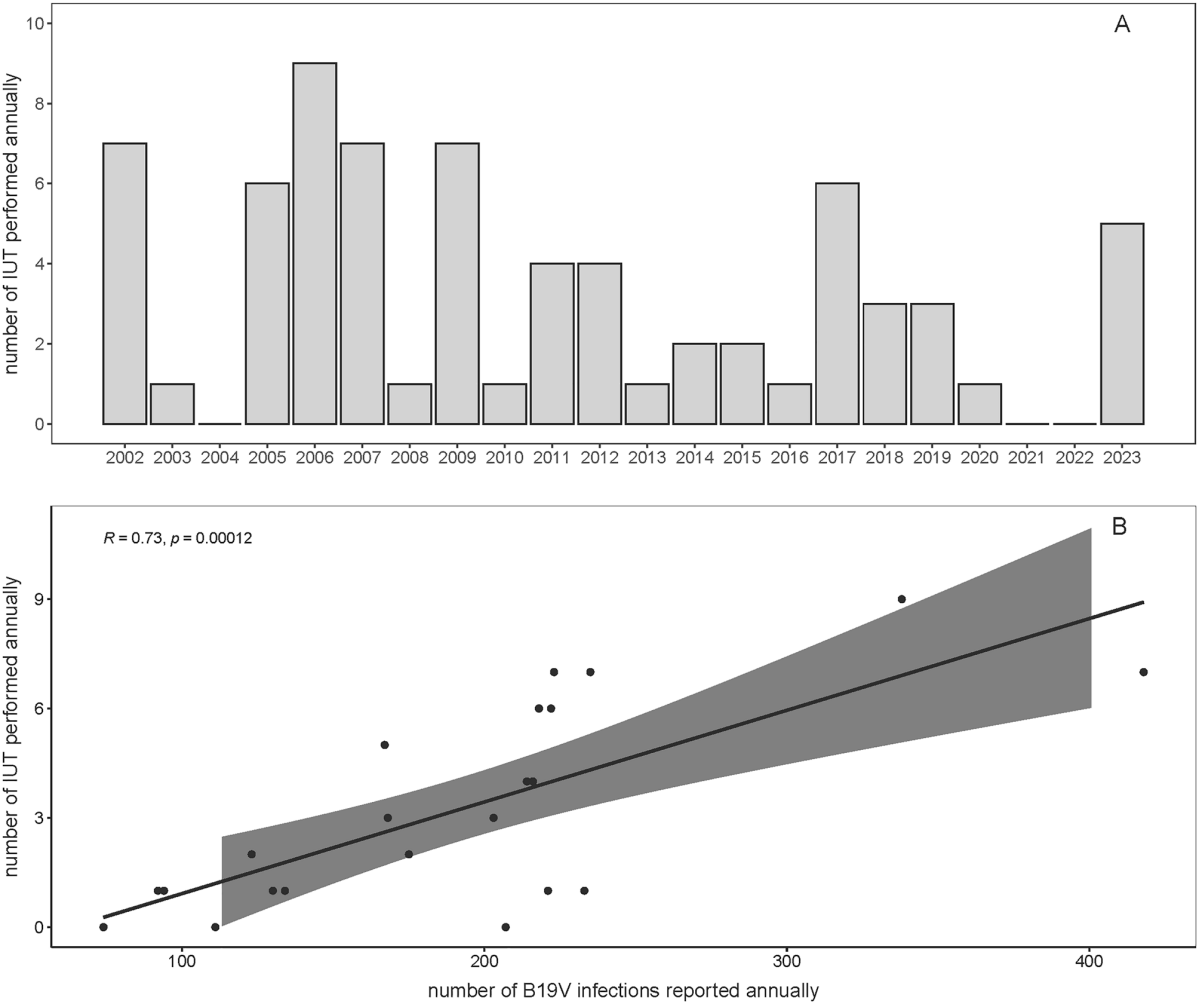
Number of annually performed IUT and correlation with reported B19V infections: (**A**) Absolute number of IUT performed annually for B19V-related fetal hydrops in the period Jan 1st, 2002–Dec 31st, 2023, in the Netherlands. (**B**) correlation between number of annually reported B19V infections by the Sentinel Surveillance and number of IUT performed annually. Gray lines indicate the confidence interval of the Pearson correlation coefficient at 95%. The p-value is the significance level of the t-test.

Data from the Dutch blood bank on the incidence of high load viremic donations showed a similar pattern to Sentinel Surveillance data, with annual peaks in viremic donations in spring (Fig. 3). For the period 2013–2019, the minimum incidence of viremic donations was 0.18 per 10.000 donations (2015) and the maximum incidence was 0.60 per 10.000 donations (2019). Figure 4a shows the annual number of IUT performed in the Netherlands for B19V-related fetal anemia in the period Jan 1st, 2002–Dec 31st, 2023. The number varies from 0 IUT per year to a maximum of 9 IUT per year.

### Cross-correlations between different time-series

Figure 4b shows the over-all correlation between Sentinel Surveillance data and IUT. Analyses of cross-correlations between Sentinel Surveillance data and IUTs at different lags showed significant correlations at different lags, with maximum lag occurring at 1 week where Sentinel Surveillance data precedes IUT data (data not shown). Cross-wavelet analysis showed similar results (data not shown).

### Near absence of B19V infections since the start of the COVID pandemic and re-emergence

In the Sentinel Surveillance data, the mean annual number of reported infections was 173 in the period 1990–2019. In the period 2020–2022 the mean annual number of reports was much lower at 92 reports per year. Data from the Dutch blood bank show that high-load viremic donors were virtually absent during the COVID-19 pandemic (Fig. 3). The incidence of viremic donations dropped to 0.01 per 10.000 donations in 2021. Also, no IUT for B19V-related fetal hydrops was performed in the period 2020–2022. In the spring of 2023, Sentinel Surveillance data showed an increasing number of B19V infections (Fig. 1), indicating the re-emergence of B19V that continues into the winter of 2023. In week 52, the 9-week running mean was 6.1. This is the highest number of reported infections since the autumn of 2019. In addition, the incidence of viremic donations increased from 0.17 per 10.000 donations in April to 1.28 per 10.000 donations in August, a level which is at least comparable with previous endemic years. In the last months of 2023, the number of B19V-positive donations is again increasing (Fig. 3). As of May 2023, 5 IUTs have been performed in 2023, of which 4 were performed in the period September–December 2023.

## Discussion

To our knowledge, our analysis considers the longest period of B19V epidemiology world-wide. Based on Sentinel Surveillance data from the Netherlands, our analyses revealed a strong 1-year periodicity of B19V epidemics over the period 1990–2014, and a weaker 4-year period of increased epidemic circulation superimposed on the annual epidemics over the period 1990–2009 (Fig. 1). This pattern of 1- and 4- year cycles has been described previously in various countries in Europe and Australia^1–4^. Interestingly, our analyses also show that these patterns have become less pronounced over the past decade, particularly the 4-year periodicity. In the pre-vaccination era, recurrent epidemics of childhood diseases such as measles, mumps, and whooping cough were also characterized by a pattern of annual epidemics and more pronounced epidemics every 2 to 5 years. These patterns have been explained by the gradual build-up of population susceptibility by demographic turnover, in particular births. A decrease in population susceptibility leads to shifts in cyclical outbreak dynamics^9–11^. This could also apply to the dynamics of B19V infections. A slowly decreasing demographic turnover as observed in the Netherlands, where the number of births has decreased from approximately 200.000 per year in 2000 to approximately 170.000 per year in the 2020s, could have contributed to the disturbance of a 4-year pattern^12^.

It is remarkable that in the blood bank data and the IUT data, B19V infections were virtually absent in the period 2020–2022, while the Sentinel Surveillance system still reported infections at low levels. The Sentinel Surveillance system receives data on diagnosed infections from a network of laboratories. B19V is not a reportable disease and symptoms are generally mild; in most cases of B19V infection there is no need for laboratory diagnosis. Therefore, the amount of laboratory-proven infections will represent only a small part of all B19V-infections in the population. The definition of diagnosed B19V infection is left to the discretion of the laboratory. In practice, laboratories diagnose and report B19V infection based on positive serology and/or PCR-proven high viral loads in blood in combination with the appropriate symptomatology, if such clinical information is available. The availability of clinical information may vary between laboratories. In this system, occasional occurrence of false-positive IgM findings, leading to incorrect reports of infection, cannot be excluded. False-positive IgM would not influence trends, but could cause a slight overestimation of infections, especially during the COVID-19 pandemic. In addition, we used data on different populations, which may have differed in timing of presentation. The Sentinel Surveillance system includes only laboratory-proven infections and thus represents symptomatic individuals presenting to healthcare providers. Data from the national blood bank report only on subclinical infections (the health check before donation will exclude symptomatic patients). IUTs are performed in symptomatic fetuses when primary maternal infection will have occurred weeks before. This may create different time lags between start of circulation of (subclinical) infection and reported infections. Nonetheless, the same trends as well as the near-absence of B19V during and after the COVID-19 pandemic are confirmed by all different sources. By making use of diverse sources of population and patient data, we have attained a complete and reliable insight into B19V epidemiology.

The annual epidemic cycle for B19V has been absent for three consecutive years. Observational data show that the epidemiology of different respiratory-transmitted viruses is differentially impacted by the COVID-19 pandemic. In the first year after COVID-19 restrictions were active in most European countries, RSV epidemics showed a lag of approximately 6 months compared to previous epidemic cycles in the first year of the pandemic^6,13–15^. Influenza followed its regular epidemic pattern, although with flattened seasonal peaks in 2021 and 2022, after an initial absence of a year^6^. B19V differs from most other respiratory-transmitted viruses such as RSV and influenza by inducing long-lasting immunity, whereas reinfections by RSV and influenza occur frequently^16,17^. For B19V, this may result in a less susceptible population when reintroduction into the population occurs. Also, it could be hypothesized that B19V must be partially reintroduced into the population from other (transcontinental) regions, in which case it would be very interesting to monitor genotype distribution after re-emergence of the infection. For RSV, it has been observed recently that following relaxation of COVID-19 restrictions in Australia, there has been a major collapse in circulating genomic lineages, with only 2 dominant RSV-A clades now circulating in large, distinct parts of Australia^18^. For B19V, the global genotype distribution differs with genotype 1 almost exclusively occurring in Europe and North America and genotype 3 being common in Africa, next to genotype 1^19,20^.

In this study, we found a clear association between epidemic years and the incidence of severe intrauterine infections. This is consistent with previous studies, in which the epidemic cycles of B19V infections were reflected in the incidence of intrauterine complications, such as intrauterine death or fetal hydrops. The risk of intrauterine death was estimated at 12 per 100.000 pregnancies in nonepidemic years to 48 in epidemic years^1,21^. Due to the absence of B19V in recent years, the number of susceptible individuals will have increased substantially. This implies that when B19V re-enters the population on a large scale, an epidemic amongst children could result in a sizeable increase in infections during pregnancy. Currently, the seasonal spring peak in 2023 as observed in this study and recently described in France and Israel, clearly indicates that the return of large-scale viral circulation has commenced^22,23^. At the time of conclusion of this study, an ongoing rise in B19V circulation is being observed, even in winter. Awareness of the potential for B19V outbreaks in the near future, requires prompt diagnostics and monitoring, to detect infections as early as possible to prevent fetal morbidity and mortality in pregnancy. This also applies to other populations susceptible to severe B19V-infection, including all persons with underlying disorders of erythropoiesis and transplant patients.

## Methods

### Data collection: Sentinel Surveillance system

National data on laboratory-proven B19V infections were obtained from the Dutch weekly Sentinel Surveillance system for the period January 1st, 1990, to December 31st, 2023. The Sentinel Surveillance system retrieves their data from a network of 21 Dutch clinical microbiological laboratories that report the absolute number of laboratory-proven B19V infections weekly to the National Institute for Public Health and the Environment (RIVM, Bilthoven, the Netherlands). Each week, the participating laboratories extract the number of patients from their records that were diagnosed with B19V-infection. Individual diagnosis can be made with serology, PCR (in case of high viral loads) or a combination of both. Because of this method, the Sentinel Surveillance system generates a representative view of the epidemiology of a given infection, but does not report on absolute incidence^24,25^.

### Dutch national blood bank (Sanquin)

In compliance with European blood banking guidelines, the Sanquin Blood Supply Foundation routinely screens donated plasma for B19V DNA to exclude donations with a high B19V load before fractionation, using a procedure as described previously that did not change during the period under study^7,26^. In short, regular donations were tested in pools of 480, and donations used for production of solvent-detergent-treated plasma were screened in pools of 96. When a pool tested positive, the pool was deconstructed to identify the donation(s) containing a high load of B19V DNA (> 1.2 × 10^6^ IU/mL). For the period January 1st, 2013, to December 31st, 2023, the monthly number of high-load B19V DNA-containing donations and the total number of donations for each month were extracted from the Sanquin records.

### IUT

All patients for whom IUT is indicated in the Netherlands are referred to the Leiden University Medical Center (LUMC). IUTs performed for fetal hydrops due to intrauterine B19V infection, as diagnosed by a positive B19V PCR on fetal blood and/or amniotic fluid, in the period January 1st, 2002, to December 31st, 2023, were included. Data on the annual number of IUT and the week in which they were performed were retracted from the obstetric records of the LUMC.

### Statistical analysis

Periodicity of the data from the Sentinel Surveillance system was studied using wavelet analysis^27,28^. Main output of the analysis is a contour plot of the relative importance of periodicities (wavelet power) in the time-periodicity plane. Before analysis, we transformed the data using a Box-Cox transformation with optimized scaling parameter, to homogenize the variance and to approximate a normal distribution^29^. Throughout, we used the Morlet wavelet^30^. Significance of the periodicities was assessed using a χ^2^ significance test^27^. Specifically, statistical significance was assessed by testing against the null hypothesis that observed periodicities are generated by a red noise process with the same autocorrelation coefficient as the data. The Pearson correlation coefficient for the Sentinel Surveillance data and IUT was calculated using the functions cor. test and ggscatter in the R package ggpubr. Cross-correlation between Sentinel Surveillance data and IUT was studied using the cross-correlation function (CCF). Our analyses of the cross-correlation and wavelets were implemented in R (version 4.3.1) using the package EnvStats to estimate the optimal parameter of the Box–Cox transformation (version 2.7.0) and the package biwavelet (version 0.20.21) to perform wavelet analysis^31,32^. Data and code are available in the supplement.

### Supplementary Information

Supplementary Information 1.

Supplementary Information 2.

Supplementary Information 3.

## Data Availability

Input data and code of the wavelet analysis are made available in the supplemental files.
