# Supplementary material for: Changing epidemiology of parvovirus B19 in the Netherlands since 1990, including its re-emergence after the COVID-19 pandemic
Source: Sci Rep. 2024 Apr 26;14:9630. doi: 10.1038/s41598-024-59582-7 (PMC11053065; doi:10.1038/s41598-024-59582-7)
Supplement: Supplementary file 2 — Supplementary Information 2. [file 41598_2024_59582_MOESM2_ESM.pdf]

```
# Script performing wavelet analysis on the parvovirus data (Figure 1)
# Upper panel running mean of BV12

# Load the packages
library(here)
library(tidyr)
library(dplyr)
library(ggplot2)
library(openxlsx)
library(biwavelet)
library(lubridate)
library(ggplotify)
library(EnvStats)
library(scales)

# read the data of parvovirus
data_file <- here("data","070324_vir weekstaten 1990_2023_tbv
wavelet.xlsx")
sheet_name <- "totaal"

week_data_parvo <- read.xlsx(data_file, sheet = sheet_name, colNames =
TRUE, rowNames = FALSE)
week_data_parvo <- week_data_parvo%>%
                   mutate(date = make_date(year = jaar) +weeks(week) ) %>%
#make date number for nice plotting
                   mutate(time = seq(1:nrow(week_data_parvo)))%>% #make
sequence number for the analysis
                   relocate(c(date,time),.before=jaar)%>%
                   rename(.,"Parvovirus"="parvo")


# visualize the data of parvovirus
plot_week_date_parvo <- week_data_parvo%>%
                        ggplot(aes(date,Parvovirus))+
                        geom_line(col="Black")+
                        theme_bw()+
                        scale_x_date(labels = date_format("%d-%m-%Y"))+
                        theme(panel.grid.major = element_blank(),
                                  panel.grid.minor = element_blank(),
                         axis.title.x = element_blank())+
                         ylab("number of B19V infections")

plot_week_date_parvo

#save the figure
ggsave("./plots/FigS1.pdf",width=10, height=6)

# transform the data with a box cox transformation optimizing the lambda
# the box cox transformation cannot handle 0 values. I add a small quantity
to 0
trans_week_data_parvo <- week_data_parvo%>%
                         mutate(Parvovirus= if_else(Parvovirus==0,
0.0001,Parvovirus))

# find the optimum lambda
lambda <- trans_week_data_parvo %>%
          transmute_at(vars(("Parvovirus")),function(x)(EnvStats::boxcox(x,
          lambda=c(-5,5),optimize=TRUE)$lambda))%>%
          slice(1)
```

```
# perform the box cox transformation with the optimized lambda
box_cox_data_parvo <- trans_week_data_parvo %>%
                      mutate_at(vars("Parvovirus"),function(x)(EnvStats
                      ::boxcoxTransform(x,lambda=lambda%>%pull())))

# plot the new transformed time series and perform wavelet on the box cox
transformed data
box_cox_data_plot <- box_cox_data_parvo%>%
                     ggplot(aes(time,Parvovirus))+
                     geom_line(col="Black")+
                     theme_bw()+
                     theme(panel.grid.major = element_blank(),
                     panel.grid.minor = element_blank())
box_cox_data_plot

# make the wavelet analysis and plot Fig 1
# select the data
data_box_cox_wt <- box_cox_data_parvo %>%
                   select(time,Parvovirus)

# compute the wavelet spectrum
wt_box_cox_parvo <- wt(data_box_cox_wt,s0=8)

#define the labels for the time axis
xticks <-which(box_cox_data_parvo$week==1)
years_labels<-box_cox_data_parvo$jaar[xticks]

#define the weeks labels
weeks_labels <-c(1,13,26,52*c(1,2,4,8))
axis.locs <-log2(weeks_labels)
yticklab <- format(2^axis.locs)

# make figure 1
pdf("./plots/Figure1.pdf",width=10, height=6)
layout_mat <- matrix(c(1,  2), nrow = 2, ncol = 1,
                     byrow = TRUE)
layout_mat
my_lay <- layout(mat = layout_mat,
                 heights = c(1, 1),
                 widths = c(2), respect =TRUE)

par(mar = c(2,4,1,0.6))
plot(week_data_parvo$time,week_data_parvo$mean_9_weeks,ylim=c(0,15),xlab=""
,ylab="9-week running mean of reported B19V
infections",xaxt='n',type='l',xaxs = "i",
      yaxs = "i",cex.axis=0.7,cex.lab=0.7)
axis(1, at=xticks,labels=F)
mtext("A", side=3, at=0,cex=0.8)

par(mar = c(4, 4, 0.1, 0.6))
plot(wt_box_cox_parvo,type = "power.corr.norm",xlab="", ylab="Period
(weeks)", xaxt='n',yaxt='n', plot.cb = FALSE, plot.phase = FALSE,legend.loc
=c(.97,0.98,0.3,0.8),cex.axis=0.7,cex.lab=0.7)
                                              axis(1, at=xticks,
labels=years_labels,cex.axis=0.7)
                                              axis(2, at=axis.locs,
labels=yticklab,cex.axis=0.7)
mtext("B", side=3, at=0,cex=0.8)
dev.off()
```
