# Supplementary material for: Changing epidemiology of parvovirus B19 in the Netherlands since 1990, including its re-emergence after the COVID-19 pandemic
Source: Sci Rep. 2024 Apr 26;14:9630. doi: 10.1038/s41598-024-59582-7 (PMC11053065; doi:10.1038/s41598-024-59582-7)
Supplement: Supplementary file 3 — Supplementary Information 3. [file 41598_2024_59582_MOESM3_ESM.pdf]

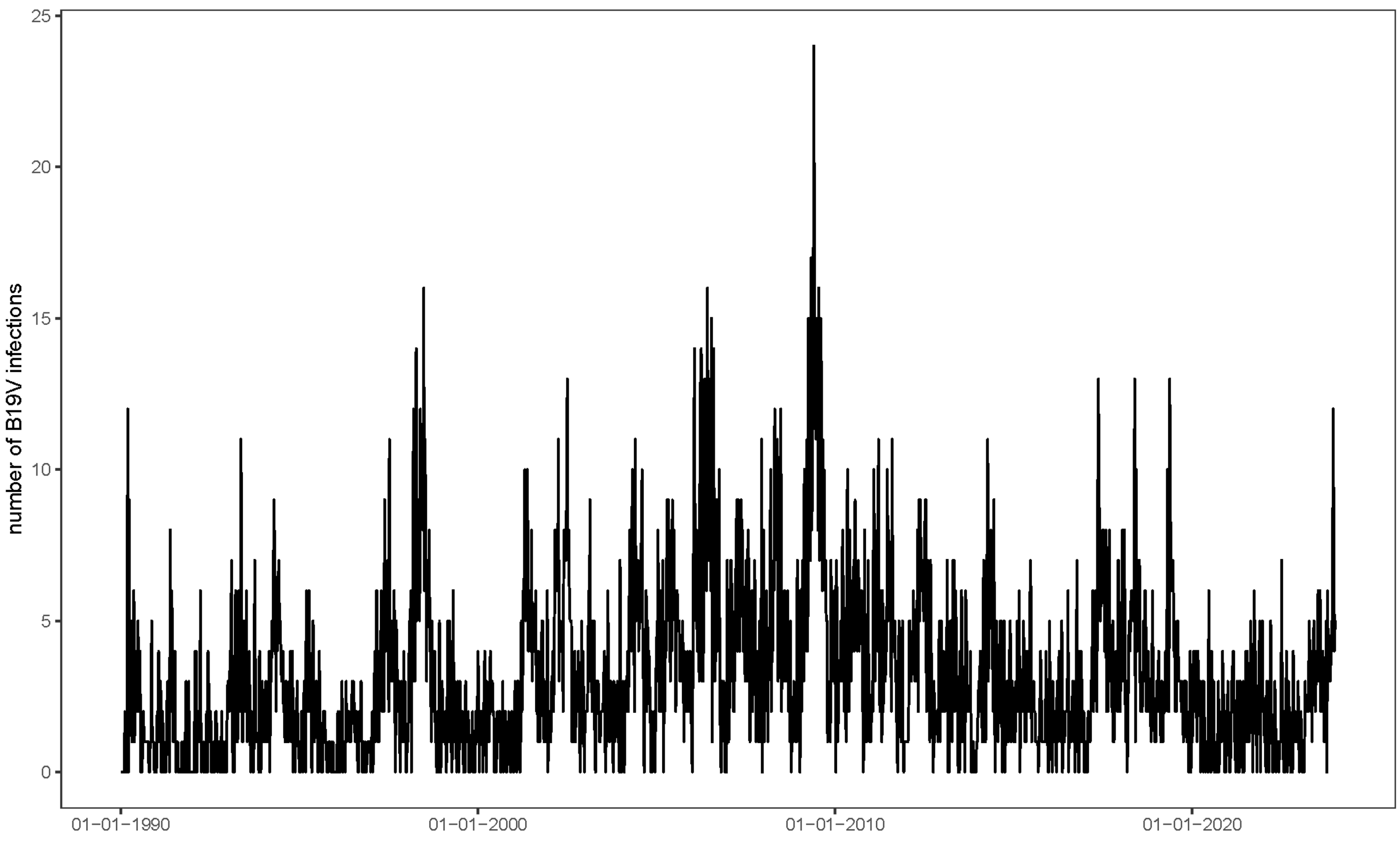

Supplementary figure S1: absolute number of weekly reported B19V infections to the Sentinel Surveillance system for the period Jan 1st 1990 – Dec 31st 2023
